# *Asiphonipponaphis*, a new genus of Hormaphidinae (Hemiptera, Aphididae) causing galls on *Distylium chinense* from China, with description of a new species

**DOI:** 10.3897/zookeys.111.1283

**Published:** 2011-06-22

**Authors:** Jing Chen, Masato Sorin, Ge-Xia Qiao

**Affiliations:** 1 Key Laboratory of Zoological Systematics and Evolution, Institute of Zoology, Chinese Academy of Sciences, No. 1 Beichen West Road, Chaoyang District, Beijing 100101, P.R.China; 2 Kogakkan University, 30–64, Sakuragaoka, Ise-shi, Mie-ken, 516–0028, Japan; 3 Graduate University of Chinese Academy of Sciences, No. 19 Yuquan Road, Shijingshan District, Beijing 100049, P.R.China

**Keywords:** *Asiphonipponaphis*, Aphididae, Hormaphidinae, new genus, new species, China

## Abstract

The aphid genus *Asiphonipponaphis* **gen. n.** from China is new to science. *Asiphonipponaphis vasigalla* **sp. n.** causing galls on *Distylium chinense* from Hunan, China is described and illustrated. Holotype and paratypes are deposited in the National Zoological Museum of China, Institute of Zoology, Chinese Academy of Sciences, Beijing, China (NZMCAS) and Kogakkan University, Japan.

## Introduction

The aphid tribe Nipponaphidini in subfamily Hormaphidinae (Hemiptera: Aphididae) occurs in East and Southeast Asia, with *Distylium* as the primary host, on which different shaped and sized galls are produced, and Fagaceae, Lauraceae and Moraceae as the secondary hosts (Ghosh 1988). Pergande (1906) described the first nipponaphidine species causing galls on *Distylium* from Japan. Since then, many gall-forming species on *Distylium* have been described (Matsumura 1917, Monzen 1934, 1954, Takahashi 1958, 1962, Hille Ris Lambers 1959, Noordam 1991, Sorin 1996). Takahashi (1962) and Sorin (1987) reviewed the species which cause galls on *Distylium* in Japan. Blackman and Eastop (1994) keyed the aphid species on *Distylium*, including free-living apterae and alatae emerging from galls. Sorin (2003) keyed the aphid species living on *Distylium racemosum* in Japan based on the galls and their detailed life cycles.

Up to now, at least eighteen species and one subspecies in twelve genera are known to induce galls on *Distylium*. Thirteen species and one subspecies are recorded on *Distylium racemosum*, viz. *Dinipponaphis autumna* (Monzen), *Indonipponaphis fulvicola* Sorin, *Metanipponaphis cuspidatae* (Essig & Kuwana), *Metanipponaphis rotunda* Takahashi, *Metanipponaphis rotunda nakijinensis* Sorin, *Metathoracaphis isensis* Sorin, *Monzenia globuli* (Monzen), *Monzenia ihai* Sorin, *Neothoracaphis yanonis* (Matsumura), *Nipponaphis distychii* Pergande, *Nipponaphis distyliicola* Monzen, *Nipponaphis loochooensis* Sorin, *Nipponaphis monzeni* Takahashi and *Quadrartus yoshinomiyai* Monzen. Five species are recorded on *Distylium stellare*, viz. *Distylaphis foliorum* (van der Goot), *Neohormaphis calva* Noordam, *Reticulaphis distylii* (van der Goot), *Schizoneuraphis gallarum* van der Goot and *Schizoneuraphis longisetosa* Noordam. In China, *Neothoracaphis yanonis* also forms galls on *Distylium chinense* (personal observation).

In this study, a new genus and a new species, *Asiphonipponaphis vasigalla* sp. n. causing galls on *Distylium chinense* is described from Hunan, China, further enriching the group of aphid species forming galls on *Distylium*.

## Materials and methods

All specimens examined in this study were collected from Jishou University (Jishou City) by X. T. Li.

Aphid terminology in this paper generally follows Ghosh (1988) and Noordam (1991). The unit of measurements in this paper is millimeters (mm).

In Table 1, the following abbreviations have been used: Ant.I, Ant.II, Ant.III, Ant.IV, Ant.Vb, for antennal segments I, II, III, IV and the base of antennal segment V, respectively; PT, processus terminalis; Ant.IIIWD, the widest diameter of antennal segment III; URS, ultimate rostral segment; BW URS, basal width of ultimate rostral segment; 2HT, second hind tarsal segment; MW hind tibia, mid-width of hind tibia; BW Cauda, basal width of cauda; AP, anal plate; GP, genital plate.

Specimen depositories: the holotype and ten paratypes of the new species are deposited in the National Zoological Museum of China, Institute of Zoology, Chinese Academy of Sciences, Beijing, China (NZMCAS), and two paratypes in Kogakkan University, Japan.

**Table 1.** Biometric data of *Asiphonipponaphis vasigalla* sp. n. (in mm).

Parts(For abbreviations see Materials and methods)		Fundatrix (n=1)	Alate vivipara (n=10)		
			Mean	Range	Standard Deviation
Length (mm)	Body length	2.036	2.394	2.204–2.492	0.098
	Body width	1.723	1.162	1.126–1.219	0.029
	Ant.I	0.057	0.062	0.052–0.073	0.008
	Ant.II	0.043	0.048	0.045–0.051	0.003
	Ant.III	0.189	0.406	0.371–0.443	0.023
	Ant.IV	/	0.183	0.156–0.203	0.015
	Ant.Vb	/	0.076	0.065–0.085	0.007
	PT	0.026	0.026	0.020–0.032	0.004
	URS	0.063	0.068	0.065–0.071	0.003
	Hind femur	0.342	0.532	0.516–0.548	0.009
	Hind tibia	0.301	0.684	0.661–0.701	0.012
	2HT	0.072	0.106	0.099–0.111	0.004
	Cauda	0.025	0.048	0.043–0.050	0.002
	BW Cauda	0.035	0.054	0.050–0.057	0.003
	Ant.IIIWD	0.038	0.051	0.048–0.056	0.002
	MW Hind tibia	0.035	0.039	0.035–0.042	0.002
	Cephalic setae	0.026	0.013	0.012–0.017	0.002
	Setae on Tergum I	0.023	0.022	0.015–0.027	0.004
	Setae on Tergum VIII	0.038	0.037	0.032–0.041	0.003
	Setae on Hind tibia	0.024	0.031	0.027–0.033	0.002
No. of setae on	Ant.I	2		1–3	
	Ant.II	2		2	
	Ant.III	0		0	
	Ant.IV	0		0	
	Ant.Vb	0		0	
	PT	0+4		0+5	
	URS	6		6	
	Tergum VIII	4		5–8	
	Cauda	9		12–19	
	Each lobe of AP	9–10		10–15	
	GP	13		39–52	
Ratio (times)	Whole antenna / Body	0.14	0.33	0.32–0.35	0.009
	Hind femur / Ant.III	/	1.32	1.21–1.48	0.084
	Hind tibia / Body	0.15	0.29	0.28–0.31	0.012
	PT / Ant.Vb	/	0.35	0.24–0.48	0.071
	URS / BW URS	1.43	1.29	1.09–1.55	0.159
	URS / 2HT	0.88	0.65	0.61–0.71	0.037
	Cauda / BW Cauda	0.74	0.90	0.80–1.01	0.071
	Cephalic setae / Ant.IIIWD	0.68	0.26	0.22–0.33	0.032
	Setae on Tergum I / Ant.IIIWD	0.61	0.43	0.29–0.53	0.078
	Setae on Tergum VIII / Ant.IIIWD	1.01	0.72	0.58–0.80	0.067
	Setae on hind tibia / MW Hind tibia	0.68	0.79	0.68–0.92	0.081

## Taxonomy

### Asiphonipponaphis gen. n.

urn:lsid:zoobank.org:act:42BF723C-37E9-4FA5-AFC1-C81FEDA1F066

http://species-id.net/wiki/Asiphonipponaphis

#### Type species.

*Asiphonipponaphis vasigalla* sp. n.

#### Etymology.

The new genus is named for the absence of siphunculi. “*A*” (Latin) means “absent”, “*sipho*” (Latin) means “siphunculi”, “*nipponaphis*” refers to its affiliation to the tribe Nipponaphidini.

#### Generic diagnosis.

In alatae, antennae 5-segmented, secondary rhinaria annular. Rostrum short, ultimate rostral segment shorter than second hind tarsal segment, with 2 pairs of primary setae and 1 pair of accessory setae. Abdomen with 5 pairs of spiracles, present on abdominal segments II–VI. Siphunculi absent in both fundatrix and emigrant alatae. Cauda knobbed, distinctly constricted at base. Anal plate bilobed. Legs normal. Tarsi 2-segmented, claws normal, first tarsal chaetotaxy of alatae 3, 3, 3. Fore wings of emigrant alatae with pterostigma narrow and long, distal margin of pterostigma forming almost a straight line with the hind margin, media unbranched, not united with cubitus, and two cubitus veins fused at base; hind wings with 2 obliques.

#### Comments.

This new genus is unique in Nipponaphidini by its peculiar galls and morphological characters. Different from other saccate galls on *Distylium*, e.g. galls of *Nipponaphis*, its galls are located on the midrib of leaves and split at the tip when mature, forming a flower-shaped opening, while galls of *Nipponaphis* are located on twigs and usually open on the lateral wall of the galls when mature. It is related to *Quadrartus* Monzen for sharing several characters in alatae, such as spiracles present on abdominal segments II–VI (i.e. 5 on each side of abdomen), distal margin of pterostigma forming almost a straight line with the hind margin, but differs from the latter as follows: antennae 5-segmented (in *Quadrartus*: 4-segmented); media of fore wings unbranched (in *Quadrartus*: once branched); siphunculi absent (in *Quadrartus*: present). It is also related to *Indonipponaphis* Ghosh & Raychaudhuri. Both of them possess 5-segmented antennae in alatae and induce galls on the midrib of leaves of *Distylium*. But the new genus differs from *Indonipponaphis* as follows: abdomen with 5 pairs of spiracles (in *Indonipponaphis*: 4 pairs); media of fore wings unbranched (in *Indonipponaphis*: once branched); siphunculi absent (in *Indonipponaphis*: present).

Taxonomy of Nipponaphidini is mostly based upon the apterae on secondary hosts. The identification of alatae is much more difficult because of the vague descriptions and limited diagnostic characters. But not all species are known by both alate and apterous morphs. Thus identification of alatae, although confusing, is still very important to the classification of Nipponaphidini. Further observations of life cycles will probably reveal more gall causers on *Distylium*,and acquisition of more morphs will facilitate the taxonomic study and clear up the confusion.

**Figures 1–7.**
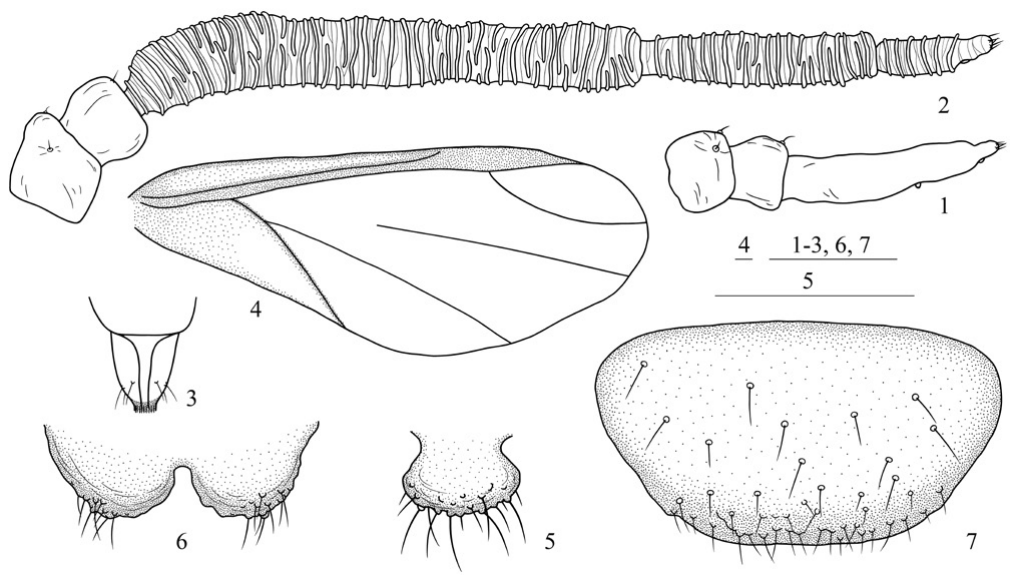
*Asiphonipponaphis vasigalla* **sp. n.** Fundatrix **1** antenna. Alate viviparous female(emigrant from galls) **2** antenna **3** ultimate rostral segment **4** fore wing **5** cauda **6** anal plate **7** genital plate. Scale bars = 0.10 mm.

### Asiphonipponaphis vasigalla sp. n.

urn:lsid:zoobank.org:act:EAA77666-5C96-4AF6-9C27-87C01F0ED006

http://species-id.net/wiki/Asiphonipponaphis_vasigalla

Figs 1 [Fig F2] –20 

#### Locus typicus.

China (Hunan, 28°17'23"N, 109°43'11"E, altitude 240 m).

#### Etymology.

The new species is named for the shape of gall. “*Vas*” (Latin) means “vase”, “*galla*” (Latin) means “gall”.

#### Description.

##### Fundatrix

**Figures 8–15. F2:**
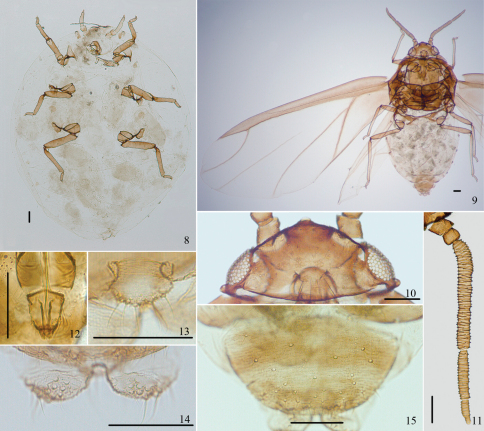
*Asiphonipponaphis vasigalla* **sp. n.** Fundatrix **8** dorsal view of body. Alate viviparous female(emigrant from galls) **9** dorsal view of body **10** dorsal view of head **11** antenna **12** ultimate rostral segment **13** cauda **14** anal plate **15** genital plate. Scale bars = 0.10 mm.

*Fundatrix*: Body oval, nearly round (Fig. 8), reddish brown and covered with thin white wax in life. For morphometric data see Table 1.

###### Mounted specimens.

Body lightly sclerotized, pale in color. Antennae, ultimate rostral segment and legs brown. Head, thorax and abdominal segments I–VII completely fused. Dorsum smooth. Abdominal tergite VIII with dense spinulose imbrications. Spiracles oval, closed, on abdominal segments II–IV, spiracular plates light brown. Dorsal setae of body little, short and pointed. Head with 1 pair of cephalic, 1 pair of spinal, 1 pair of pleural and 1 pair of marginal setae; thoracic nota each with 1 pair of spinal, 1 pair of pleural and 2 pairs of marginal setae; abdominal tergites I–VI each with 1 pair of spinal, 1 pair of pleural and 1 pair of marginal setae; tergite VII with 1 pair of marginal setae; tergite VIII with 4 setae. Cephalic setae, marginal setae on abdominal tergite I and setae on tergite VIII 0.68 times, 0.61 times and 1.01 times as long as widest diameter of antennal segment III, respectively. Front straight. Eyes 3-faceted. Antennae 3- or 4-segmented (Fig. 1); 0.14 times as long as body. Length in proportion of segments I–III: 35 : 26 : 100+16, respectively. Processus terminalis 0.16 times as long as base of the segment III; very thin, basal width 0.47–0.57 times as long as apical width of base of the segment III. Setae on antennae sparse. Segments I–III each with 2, 2, 0+0 setae, respectively. Processus terminalis with 4 apical setae. Primary rhinaria small, round, protuberant and placed wide apart. Rostrum short and thick, not reaching mid-coxae. Ultimate rostral segment blunt wedge-shaped, 1.43 times as long as its basal width, 0.88 times as long as second hind tarsal segment; with 2 pairs of primary setae and 1 pair of accessory setae. Legs normal. Trochanters and femora fused. Hind trochanter and femur 2.11 times as long as the base of antennal segment III, hind tibia 0.15 times as long as body. Setae on legs sparse, fine and pointed. Setae on hind tibia 0.68 times as long as its mid-diameter. First tarsal chaetotaxy: 2, 2, 2. Siphunculi absent. Cauda, anal plate and genital plate with dense spinulose imbrications. Cauda knobbed, indistinctly constricted at base, 0.74 times as long as its basal width, with 9 setae. Anal plate bilobed, each with 9 or 10 setae. Genital plate broad round, with 2 anterior setae and 11 setae along the posterior margin. Two gonapophyses each with 3 or 4 short setae.

##### Alate viviparous females

(emigrants from galls): Body oval (Fig. 9), cephalothorax black, abdomen dark reddish brown and pterostigma black in life, wings flat in repose. For morphometric data see Table 1.

###### Mounted specimens.

 Head, thorax, antennae, ultimate rostral segment, legs and genital plate brown, abdominal tergites VII–VIII with brown broad transverse bands, forewing veins and pterostigma brown, the other parts of body pale. Dorsum of head with sparse imbrications, tibiae, tarsi, venter of femora and abdominal tergites VI–VIII with dense spinulose imbrications. Spiracles oval, closed, on abdominal segments II–VI, spiracular plates brown. Dorsal setae of body short and pointed, on light brown seta-bearing sclerites. Head with 10–25 dorsal setae between antennae and 15–20 dorsal setae between eyes; pronotum with 1 pair of spinal, 1 pair of pleural and 2 pairs of marginal setae; mesonotum with 19–30 setae; abdominal tergite I with 5–8 spino-pleural and 1 pair of marginal setae; abdominal tergite II with 4–9 spino-pleural and 1 pair of marginal setae; abdominal tergite III with 6–8 spino-pleural and 1 pair of marginal setae; abdominal tergites IV–V each with 5–8 spino-pleural and 1 pair of marginal setae; abdominal tergites VI–VII each with 4–6 spino-pleural and 1 pair of marginal setae; tergite VIII with 5–8 setae. Cephalic setae, marginal setae on abdominal tergite I and spinal setae on tergite VIII 0.22–0.33 times, 0.29–0.53 times and 0.58–0.80 times as long as widest diameter of antennal segment III, respectively.

Head (Fig. 10): Front protuberant. Eyes compound. Antennae 5-segmented (Figs. 2, 11), with dense spinulose imbrications on segments III–V; 0.32–0.35 times as long as body. Length in proportion of segments I–V: 15 : 12 : 100 : 45 : 19+7, respectively. Processus terminalis 0.24–0.48 times as long as base of the segment V. Setae on antennae sparse. Segments I–V each with 1–3, 2, 0, 0, 0+0 setae, respectively. Processus terminalis with 5 apical setae. Primary rhinaria small, round and ciliated. Segments III, IV and base of segment V each with 37–44, 16–21, 6–9 annular secondary rhinaria, respectively. Rostrum short, not reaching mid-coxae. Ultimate rostral segment blunt wedge-shaped (Figs. 3, 12), 1.09–1.55 times as long as its basal width, 0.61–0.71 times as long as second hind tarsal segment; with 2 pairs of primary setae and 1 pair of accessory setae.

Thorax: Legs normal. Trochanters and femora fused. Hind trochanter and femur 1.21–1.48 times as long as antennal segment III, hind tibia 0.28–0.31 times as long as body. Setae on legs dense, fine and pointed. Setae on hind tibia 0.68–0.92 times as long as its mid-diameter. First tarsal chaetotaxy: 3, 3, 3. Dorso-apical setae on second hind tarsal segments expanded at apices. Empodial setae pointed, exceeding tip of claws. Fore wings (Figs. 4, 9) with pterostigma narrow and long, distal margin of pterostigma forming almost a straight line with the hind margin, media unbranched, not united with cubitus, and two cubitus veins fused at base; hind wings with 2 obliques.

Abdomen: Siphunculi absent. Cauda, anal plate and genital plate with dense spinulose imbrications. Cauda knobbed, distinctly constricted at base (Figs. 5, 13), 0.80–1.01 times as long as its basal width, with 12–19 setae. Anal plate bilobed (Figs. 6, 14), each with 10–15 setae. Genital plate broad round (Figs. 7, 15), with 39–52 setae. Two gonapophyses each with 3–9 short setae.

#### Specimens examined.

Holotype: alate viviparous female, **CHINA**: Hunan (Jishou City, 28°17'23"N, 109°43'11"E, altitude 240 m), 21 Apr. 2010, No. Y8974–1-8, on *Distylium chinense*, coll. X. T. Li (NZMCAS). *Paratypes*: 1 fundatrix and 11 alate viviparous females, with the same collection data as holotype.

#### Taxonomic notes.

 The new species is similar to *Indonipponaphis fulvicola* Sorin, but differs from the latter as follows: Fundatrix: body larger, 2.036 mm long (the latter: about 1.250 mm long); first tarsal chaetotaxy: 2, 2, 2 (the latter: 3, 3, 2); siphunculi absent (the latter: present); cauda knobbed (the latter: round). Alatae from galls: base of antennal segment V with 6–9 secondary rhinaria (the latter: 11–14); abdomen with 5 pairs of spiracles (the latter: 4 pairs); first tarsal chaetotaxy: 3, 3, 3 (the latter: 3, 3, 2); media of fore wings unbranched (the latter: once branched); siphunculi absent (the latter: present); cauda knobbed (the latter: round).

#### Host plant.

*Distylium chinense*.

#### Biology.

**Figures 16–20.**
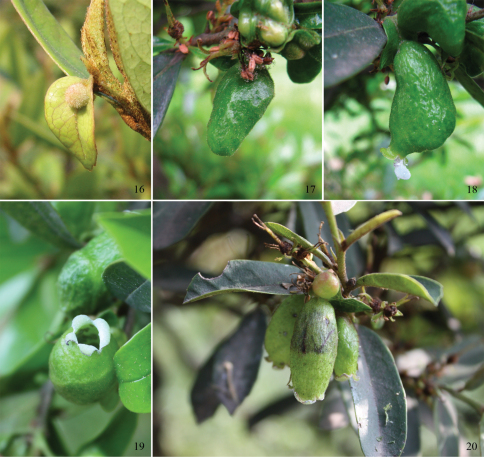
Galls of *Asiphonipponaphis vasigalla* **sp. n.** on *Distylium chinense* **16** small spherical gall on young leaf **17** large long saccate gall **18** large honeydew droplet coated with wax is being expelled through the opening **19** flower-shaped opening at the tip of gall **20** vase-shaped mature galls.

The aphids live in galls on the upper side of leaves of *Distylium chinense*. In early March, small galls start to grow on young leaves, often rise from or near the midrib, spherical, pale green, sometimes with a pinkish tinge due to the dense soft hairs on the surface (Fig. 16). Usually one leaf bears only one gall. After about 30 days, the galls when fully developed are large, long, saccate, approximately 3.2 cm in length and 1.1 cm in diameter (Fig. 17). Later, they split at the tip, forming a flower-shaped opening (Figs. 19, 20), through which large honeydew droplets coated with much wax are expelled (Fig. 18). The galls are vase-shaped when mature (Fig. 20). The alate viviparous females mature in the galls in late April and fly to an unknown secondary host.

## Supplementary Material

XML Treatment for Asiphonipponaphis

XML Treatment for Asiphonipponaphis vasigalla
